# Supplementary figures and images for: INX-18 and INX-19 play distinct roles in electrical synapses that modulate aversive behavior in Caenorhabditis elegans
Source: PLoS Genet. 2019 Oct 28;15(10):e1008341. doi: 10.1371/journal.pgen.1008341 (PMC6837551; doi:10.1371/journal.pgen.1008341)

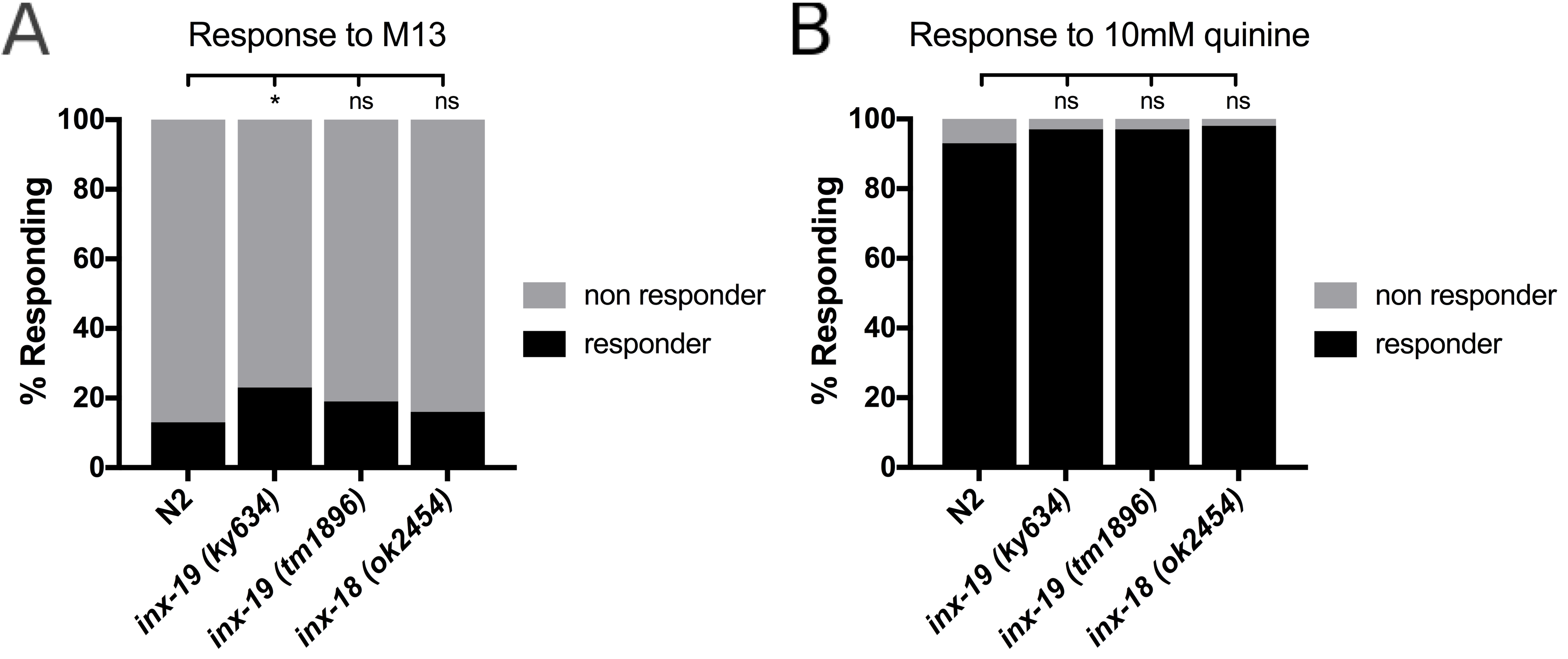

Supplement: S1 Fig — A) Inx-19(tm1896) and inx-18(ok2454) mutant animals respond at N2 (wild-type) levels when presented with M13 buffer, while inx-19(ky634) animals respond slightly more than wild-type animals. N2 = 13%, n = 330; inx-19(ky634) = 23%, n = 120, p = 0.012; inx-19(tm1896) = 19%, n = 210, p = 0.07; inx-18(ok2454) = 16%, n = 160, p = 0.33. B) Inx-19(ky634), inx-19(tm1896), and inx-18(ok2454) mutant animals respond at wild-type levels when presented with 10 mM quinine. N2 = 93%, n = 330; inx-19(ky634) = 97%, n = 120, p = 0.18; inx-19(tm1896) = 97%, n = 210, p = 0.03; inx-18(ok2454) = 98%, n = 120, p = 0.02. (TIF) [file pgen.1008341.s001.tif]

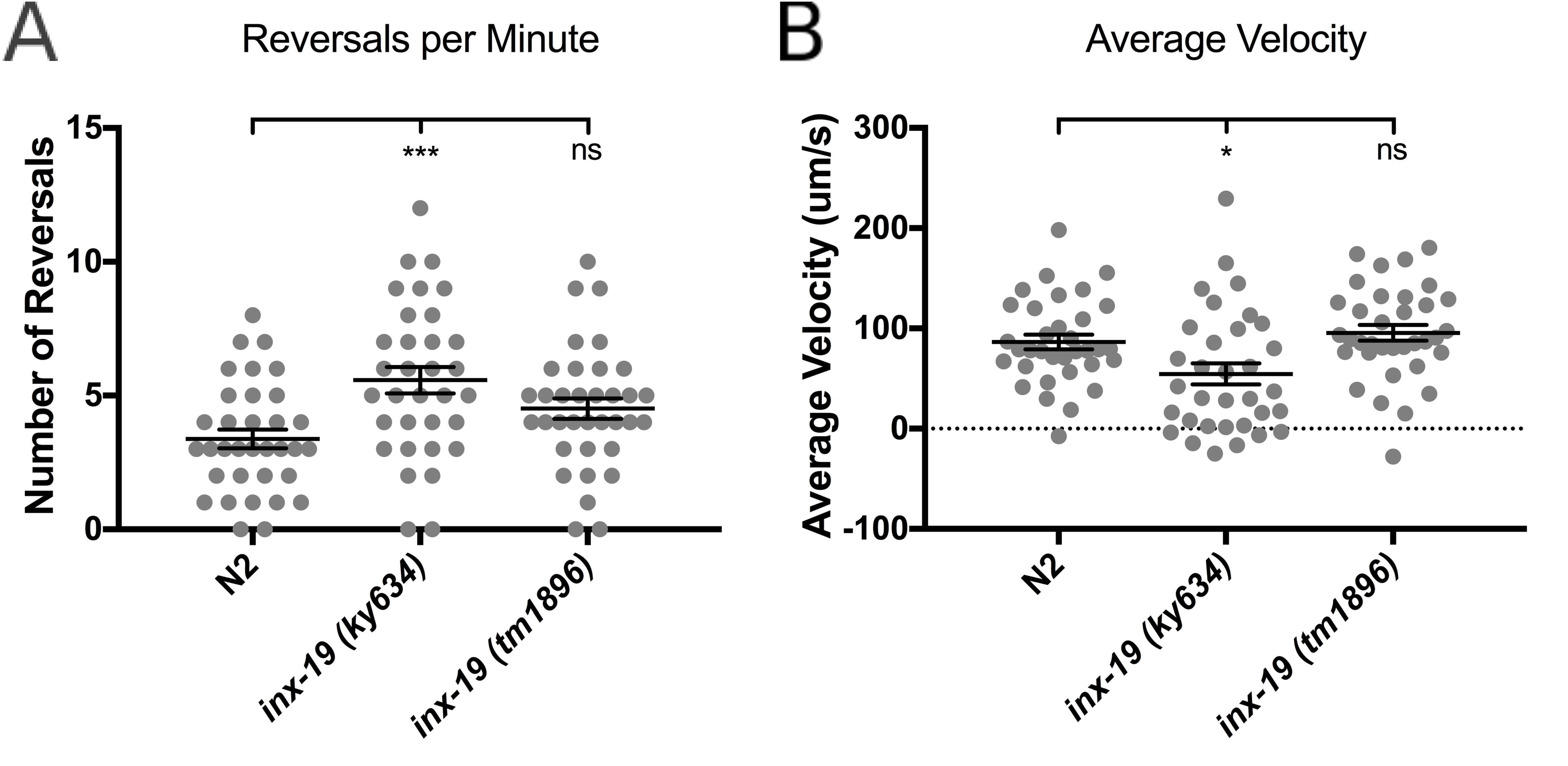

Supplement: S2 Fig — A) Inx-19(ky634) mutant animals reverse more frequently than N2 (wild-type) animals. Number of reversals were counted from a one-minute video. One-way ANOVA between three groups showed significant differences (F[2,99] = 6.943, p = 0.0015, α = 0.05), and Dunnett’s multiple comparison test showed that N2 (n = 34) differed from inx-19(ky634) (n = 33, p = 0.0006) but not inx-19(tm1896)(n = 35, p = 0.097). B) Inx-19(ky634) mutant animals have lower average movement velocity than N2 animals. One-way ANOVA between three groups showed significant differences (F[2,99] = 6.089, p = 0.003, α = 0.05), and Dunnett’s multiple comparison test showed that N2 (n = 34) differed from inx-19(ky634) (n = 33, p = 0.021) but not inx-19(tm1896)(n = 35, p = 0.677). Each data point represents a single worm and error bars are ±SEM. (TIF) [file pgen.1008341.s002.tif]

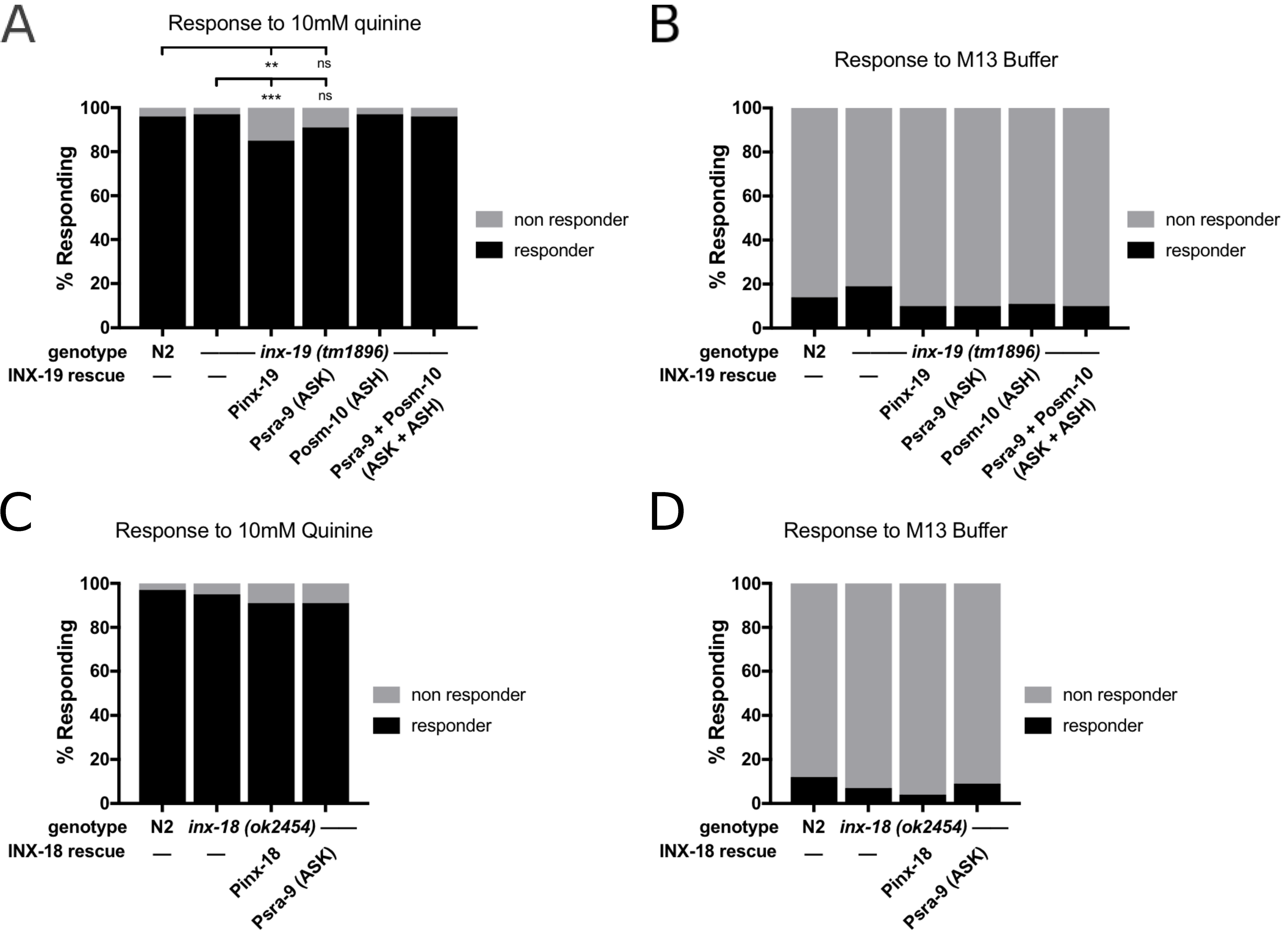

Supplement: S3 Fig — A) Inx-19(tm1896) animals carrying rescue transgenes behaved like N2 (wild-type) animals when presented with M13 buffer. N2 = 14%, n = 220; inx-19(tm1896) = 19%, n = 210; inx-19;Pinx-19::inx-19cDNA = 10%, n = 100; inx-19;Psra-9::inx-19cDNA = 10%, n = 100; inx-19;Posm-10::inx-19cDNA = 11%, n = 110; inx-19;Psra-9::inx-19cDNA; Posm-10::inx-19cDNA = 10%, n = 110. B) Inx-18(ok2454) animals carrying rescue transgenes behaved like N2 animals when presented with M13 buffer. N2 = 12%, n = 120; inx-18(ok2454) = 7%, n = 120; inx-18;inx-18gDNA = 4%, n = 100; inx-18;Psra-9::inx-18cDNA = 9%, n = 120. C) Inx-19(tm1896) animal carrying neuron-specific transgenes behaved like N2 animals when presented with 10 mM quinine, but expression of inx-19 cDNA using the native promoter reduced the responses to 10 mM quinine below wild-type levels. N2 = 96%, n = 220; inx-19(tm1896) = 97%, n = 210; inx-19;Pinx-19::inx-19cDNA = 85%, n = 100, p = 0.002 vs N2, p = 0.0004 vs inx-19; inx-19;Psra-9::inx-19cDNA = 91%, n = 100, p = 0.10 vs N2, p = 0.04 vs inx-19; inx-19;Posm-10::inx-19cDNA = 97%, n = 110, p = 0.76 vs N2, p>0.99 vs inx-19; inx-19;Psra-9::inx-19cDNA; Posm-10::inx-19cDNA = 96%, n = 110, p>0.99 vs N2, p = 0.74 vs inx-19. D) When expressing inx-18 cDNA under the native promoter or in ASK, inx-18(ok2454) animals behaved like wild-type when presented with 10 mM quinine. N2 = 97%, n = 120; inx-18(ok2454) = 95%, n = 120; inx-18;inx-18gDNA = 91%, n = 100; inx-18;Psra-9::inx-18cDNA = 91%, n = 120. (TIF) [file pgen.1008341.s003.tif]
