# Supplementary material for: INX-18 and INX-19 play distinct roles in electrical synapses that modulate aversive behavior in Caenorhabditis elegans
Source: PLoS Genet. 2019 Oct 28;15(10):e1008341. doi: 10.1371/journal.pgen.1008341 (PMC6837551; doi:10.1371/journal.pgen.1008341)
Supplement: Attachment — Submitted filename: _Voelker-et-al_Responses.docx [file pgen.1008341.s005.docx]

**Reviewer comments:**

**Reviewer #1:**

*In this manuscript, Lisa et al investigate the physiological function of electrical synapses in behavioral modulation in C. elegans. The authors have combined genetic manipulation, morphological analysis, behavioral test and imaging analysis to clearly show that the electrical synapses, including both INX-18 and INX-19, between two sensory neurons (ASH and ASK) modulate the quinine induced aversive response in ASH by controlling cGMP redistribution. This article provide a novel insight into the molecular basis for the the bias communication of intracellular signal cGMP between sensory neurons. Also, the article show that electrical synapses with different components have distinct roles in controlling cGMP levels in ASK and ASH. All experiments are well designed, and result provide strong support for their conclusion, so I am happy to support the paper published on Plos Genetics with minor changes.*

We thank reviewer’s support for publication. We have addressed all concerns in the revised manuscript, as detailed below.

*Minor Comments:
1: The result shows that electrical synapses composed by inx-19 is permeable for cGMP, but not Ca2+. Is it possible the molecular different between inx-18 and inx-19 contribute to the permeability difference? Please comment.*

We agree with reviewer #1 that molecular differences between the two innexins may contribute to the permeability difference. To clarify this point, we have added the following line to the third paragraph of the revised manuscript: “The composition of electrical synapses is key in determining their permeability of small molecules (such as Ca^2+^ and cGMP), and heterotypic composition is a major cause of rectification.”

*2: The figure legends have lots of redundant information, especially for statistical analysis. It is better to put them in the method.*

We have moved redundant information to the methods. We left in values that were specific for each experiment/strain such as percent, n, and p-value.

*3: Unless I missed, I did not find which transgenic animals were used for fig 3.*

We thank review #1 for pointing out our oversight and have corrected this in the manuscript. The animals used for fig 3 were wild type animals.

*4: The ASH promoter (osm-10) used in the paper also weakly expresses in ASI neuron, so it is possible that fluorescence disturb from ASI mess up the imaging single from ASH, especially for INX-19 puncta calculation in fig 3. Although I believe that the strong INX-19 fluorescence signalling comes from ASH based on my experience, it will be much clearer if the author clarify it in the manuscript.*

To make sure that INX-19 fluorescence was from the ASH neurons, we traced ASH axons using soluble BFP. For figure 3, we only analyzed puncta in ASH axons that expressed BFP, increasing the likelihood for recognizing ASH INX-19. Additionally, ASI is not known to make electrical synapses with either ASH or ASK and it appears to make only one electrical synapse in the nerve ring (between ASIR and ASIL). We have updated the methods to clarify this point “The number of INX-18 and INX-19 puncta within TagBFP-expressing ASH and ASK axons in the nerve ring was counted.”

*5: In Fig 6 A and B, the marker of Y-axis, the change in fluorescence, is not clear. I believe it should be the percentage change based on GCaMP intensity calculation if I am correct. So please clarify it.*

We have updated the Y axis label as the reviewer #1 suggested.

**Reviewer #2:**

*This manuscript from the Bai group addresses the role of electrical synapses in avoidance behavior. The manuscript investigates two genes inx-19 and inx-19, which are expressed in two different sensory neurons ASH and ASK. The authors postulate that expression of the two proteins in necessary in the different sensory neurons for the communication to occur. This is an excellent manuscript and I recommend the publication of the manuscript without any changes! The experiments are well conducted with the appropriate controls.*

We thank the reviewer #2 for the positive evaluation.

*I do have a couple of queries that I hope the authors can address:
1. the inx-19 allele (ky634) seems like a pleiotropic allele. IS the sensory responses to quinine in this allele due to its movement defects. Have the authors tested any other deterrents and do they observe similar locomotory defects.*

We have not tested other deterrents. Because we suspect that the increased reversal rate seen in the *ky634* allele may be pleiotropic and could contribute to the heightened responses to quinine, we have avoided using the *ky634* allele in this study beyond the intial experiments. We agree with the reviewer that it would be interesting to examine other deterrents on the *ky634* worms in the future.

*2. Does the inx-19 puncta also show differences in the two different alleles ky634 and tm1896? This is not a suggestion of a new experiment but just a curiosity to know if different alleles elicit differences in expression.*

This is an interesting point. We have not done this as we stopped using the *ky634* strain due to the locomotory changes of the *ky634* allele. Given that the *ky634* mutation produces a point mutation in the extracellular loop, it is possible that the mutant INX-19 protein trafficks correctly although it lacks normal function.

*3. Have the authors tested different concentrations of the quinine on the double mutant inx-19;inx-18?*

We did initial tests on single mutants with different concentrations of quinine (1.5mM and 2mM in addition to 1mM) in order to optimize the experimental method. However, we did not try different concentrations of quinine on the double mutant. We agree with the Reviewer #2, given that INX-18 and INX-19 have different roles, it is possible that a detailed analysis with various quinine concentrations could uncover behavioral differences between double and single mutants. To be specific about our results, we have modified the text as the following “our results suggest that the main function of INX-18 is carried out through its regulation of INX-19, as the inx-18 and inx-19 mutants do not show additive responses to 1 mM quinine.”
